# The mediating role of self-care in the relationship between eHealth literacy and quality of life among adolescents with type 1 diabetes

**DOI:** 10.1186/s12887-026-07050-8

**Published:** 2026-06-22

**Authors:** Afsaneh Ranaei, Elaheh lael-Monfared, Nooshin Peyman

**Affiliations:** 1 Department of Health Education and Health Promotion, School of Health, Mashhad University of Medical Sciences, Mashhad, Iran; 2 Student Research Committee, Mashhad University of Medical Sciences, Mashhad University of Medical Sciences, Mashhad, Iran; 3 Social Determinants of Health Research Center, Mashhad University of Medical Sciences, Mashhad, Iran

**Keywords:** Health Literacy, Digital, Diabetes Mellitus, Type 1, Self-Management, Health-Related Quality of Life, Adolescent, Digital Health

## Abstract

**Background:**

Type 1 diabetes is a common chronic disease in adolescents, requiring continuous self-care and attention to quality of life. With the rise of digital health technologies, eHealth literacy may play a key role in promoting self-care behaviors and well-being. This study aimed to investigate the relationships between eHealth literacy, self-care behaviors, and quality of life in adolescents with type 1 diabetes.

**Methods:**

In this descriptive-analytical cross-sectional study, 250 adolescents with type 1 diabetes completed validated questionnaires assessing Electronic Health Literacy (eHEALS), diabetes-specific self-care behaviors (SMOD-A), and health-related quality of life (Diabetes Quality of Life for Youth [DQOLY]). Data were analyzed using descriptive statistics, Spearman’s correlation, and multiple linear regression.

**Results:**

Participants (mean age = 14.36 years; 54.8% male) mostly had low eHealth literacy (81.2%). eHealth literacy correlated positively with self-care (*r* = 0.43) and quality of life (*r* = 0.36), while self-care was strongly related to quality of life (*r* = 0.51; *p* < 0.01). Regression models explained 35% of the variance in self-care and 29% in quality of life. Mediation analysis confirmed a significant indirect effect of eHealth literacy on quality of life through self-care behaviors (β = 0.219, *p* < 0.05).

**Conclusions:**

Enhancing eHealth literacy plays a key role in improving self-care behaviors among adolescents with type 1 diabetes, which is directly associated with better quality of life. The findings of this study clearly highlight the importance of promoting eHealth literacy as an essential component of targeted, technology-based educational interventions to improve diabetes management in this age group.

## Introduction

Type 1 Diabetes Mellitus (T1DM) is a chronic autoimmune condition that primarily affects children and adolescents. It is characterized by the destruction of pancreatic beta cells and lifelong dependence on insulin [1]. According to the International Diabetes Federation, more than 1.1 million individuals under 20 years of age worldwide live with T1DM, and its prevalence continues to rise in many countries, including Iran [2]. Recent epidemiological studies in Iran also report a notable increase in the incidence of T1DM among adolescents, creating growing challenges for the healthcare system.

Effective management of T1DM requires consistent engagement in several self-care behaviors, including regular blood glucose monitoring, insulin administration, adherence to dietary recommendations, and routine physical activity [3]. Inadequate adherence to these behaviors is associated with acute and long‑term complications and can negatively affect quality of life [4]. Adolescence is a developmental period marked by significant biological, cognitive, and social changes. It is also a critical stage for the formation of identity, behavioral independence, and personal responsibility [5]. During this period, some adolescents may struggle to follow their diabetes care plans consistently. These challenges often stem from concerns about appearing different from peers, fear of rejection, a strong desire for independence, and changes in family and social relationships [4]. Therefore, adolescence represents a particularly sensitive stage in diabetes management.

The rapid growth of digital technologies has created new opportunities to support diabetes self‑management. Adolescents are among the most frequent users of digital tools such as smartphones, mobile health applications, and social media platforms [6]. However, access alone is not sufficient to improve health outcomes. eHealth literacy—the ability to locate, understand, evaluate, and use digital health information—plays a crucial role in enabling effective use of these resources [6–8].

Health‑related quality of life (HRQoL) is an important outcome for adolescents with diabetes and is closely linked to the adequacy of self‑care behaviors [6, 7, 9]. Evidence suggests that higher eHealth literacy may improve HRQoL by strengthening self‑efficacy and enhancing self‑care practices [7, 8]. Studies from South Korea and Taiwan have reported positive associations between eHealth literacy, better glycemic control, and improved diabetes self-management [7]. Although findings from Iran are still limited, several studies have shown similar links between health literacy and quality of life in diabetic populations [7].

Most existing studies have focused on adults with type 2 diabetes. Far fewer have examined the role of eHealth literacy among adolescents living with type 1 diabetes [3]. Considering the rising prevalence of T1DM and adolescents’ increasing reliance on digital health tools, it is important to simultaneously assess eHealth literacy, self-care behaviors, and quality of life in this population. In particular, self-care behaviors may act as a mediator through which eHealth literacy influences HRQoL [7, 9]. Therefore, this study aimed to investigate the relationships among eHealth literacy, self-care behaviors, and quality of life in adolescents with type 1 diabetes.

## Methods

### Study design and setting

This descriptive–analytical cross-sectional study was conducted at the Diabetes Association of Mashhad, Iran, between April and June 2025. The study examined the relationships among eHealth literacy, self care behaviors, and health related quality of life (HRQoL) in adolescents with type 1 diabetes mellitus (T1DM).

### Participants

The study population consisted of adolescents aged 12–18 years with a confirmed diagnosis of T1DM who were registered members of the Diabetes Association of Mashhad in eastern Iran.

The inclusion criteria were: (1) physician-confirmed diagnosis of T1DM, (2) age between 12 and 18 years, (3) ability to read and complete the Persian self-report questionnaires, and (4) willingness to participate with written informed consent provided by both the adolescent and a parent or legal guardian.

Participants were excluded if they submitted incomplete questionnaires, withdrew from the study at any stage, or were unable to complete the questionnaires independently [10].

### Sample size and sampling

A list of eligible adolescents was obtained from the Diabetes Association of Mashhad. Each individual was assigned a unique identification code. Simple random sampling was then performed using a random numbers table to select participants.

The required sample size was estimated using the formula for correlation studies based on parameters reported in a previous study by Kermansaravi et al. Assuming a Type I error of 0.05, a statistical power of 0.80, and an expected correlation coefficient between 0.20 and 0.25, the minimum required sample size was estimated to be approximately 188 participants. To account for potential non-response or incomplete data, the sample size was increased to 250.

A total of 250 adolescents were invited to participate. All participants completed the questionnaires and were included in the final analysis, resulting in a response rate of 100%.

### Study variables

The primary variables examined in this study were eHealth literacy (independent variable), self-care behaviors (mediator), and health-related quality of life (outcome). Demographic and clinical characteristics included age, gender, duration of diabetes, type of insulin therapy, smartphone ownership, internet access at home, family history of diabetes, parental education level, place of residence, and perceived family socioeconomic status.

### Data collection instruments

#### Demographic questionnaire

A researcher-developed demographic questionnaire was used to collect information on age, gender, duration of diabetes (≤ 3 years or > 3 years), type of insulin therapy (multiple daily injections or insulin pump), smartphone ownership, internet access at home, family history of diabetes, parental education level, place of residence, and perceived family socioeconomic status.

#### eHealth Literacy Scale (eHEALS)

eHealth literacy was measured using the Persian version of the eHealth Literacy Scale (eHEALS), adapted by Rasouli et al. This 8-item instrument assesses individuals’ perceived ability to locate, understand, evaluate, and apply electronic health information. Items are scored on a 5-point Likert scale, yielding total scores ranging from 8 to 40. Higher scores indicate greater perceived eHealth literacy [11].

#### Self-Management of Diabetes in Adolescents (SMOD-A)

Self-care behaviors were assessed using the Persian version of the Self-Management of Diabetes in Adolescents (SMOD-A). This instrument consists of 50 items covering five domains: collaboration with parents, diabetes self-care activities, problem solving, diabetes communication, and diabetes goals. Items are rated on a 4-point Likert scale, and higher total scores indicate better diabetes self-management [12].

#### Diabetes Quality of Life for Youth (DQOLY)

HRQoL was measured using the Persian version of the Diabetes Quality of Life for Youth (DQOLY) questionnaire. The instrument evaluates multiple domains, including life satisfaction, treatment impact, activity limitations, worries, and parental control. Items are rated on a 5-point Likert scale, with higher scores indicating better health-related quality of life [10]. The internal consistency of the instruments in the present study was assessed using Cronbach’s alpha.Details of the instruments, including the number of items, scoring methods, interpretation, and reliability indices, are summarized in Table 1.

**Table 1:** Characteristics and Psychometric Properties of Study Instruments

Instrument	Number of items	Score range	Scoring method	Interpretation	Reliability (Cronbach’s α)
eHEALS	8	8–40	5-point Likert (1–5)	≤24 = low25–31 = moderate≥32 = high	0.93
SMOD-A	50	50–200	4-point Likert (1–4)	Higher scores indicate better self‑care behaviors	0.77
DQOLY	52	0–188	5-point Likert (0–4)	Higher scores indicate better HRQoL	0.87

### Data collection procedure

After obtaining approval from Mashhad University of Medical Sciences and coordinating with the Diabetes Association of Mashhad, selected adolescents and their parents were invited to participate. The objectives and procedures of the study were explained to them, and written informed consent was obtained from both adolescents and their parents or legal guardians.

Participants completed the questionnaires individually in a supervised setting at the Diabetes Association. Completing the questionnaires required approximately 20–25 min.

### Statistical analysis

Data were analyzed using SPSS version 16 (IBM Corp., Armonk, NY, USA). Descriptive statistics, including means, standard deviations, frequencies, and percentages, were used to summarize demographic characteristics and study variables.

Because some variables were not normally distributed according to the Kolmogorov–Smirnov test, Spearman’s rank correlation coefficient was used to examine relationships among eHealth literacy, self-care behaviors, and HRQoL.

Multiple linear regression analyses were conducted to identify predictors of self-care behaviors and HRQoL. Independent variables included age, eHealth literacy, family history of diabetes, smartphone ownership, and type of insulin therapy.

To test the hypothesized mediating role of self-care behaviors in the relationship between eHealth literacy and HRQoL, mediation analysis was performed using the PROCESS macro for SPSS (Model 4). In this model, eHealth literacy was specified as the independent variable, self-care behaviors as the mediator, and HRQoL as the outcome variable. Because the study employed a cross-sectional design, mediation results were interpreted as indirect associations rather than causal pathways.

The indirect effect was estimated using nonparametric bootstrapping with 5,000 resamples, and percentile bootstrap confidence intervals were calculated. An indirect effect was considered statistically significant if the 95% confidence interval did not include zero.

Subgroup interaction analyses were not included in the primary analytical plan because the study was not powered to detect interaction effects. Therefore, the main analyses were conducted on the full sample [13, 14].

## Results

A total of 250 adolescents with type 1 diabetes participated, with a mean age of 14.36 ± 1.65 years; 54.8% were male and 45.2% female. Most participants used multiple daily insulin injections (82.0%), owned a smartphone (75.6%), and had internet access at home (52.0%). A family history of diabetes was present in 21.6%. Most mothers (78.4%) and fathers (64.4%) had a university-level education, and the majority lived in urban areas (64.8%) with middle socioeconomic status (69.2%) (Table 2).

**Table 2:** Demographic Characteristics of Participants (*n* = 250)

Variable	Category	Frequency (*n*)	Percentage (%)
Age (years), mean ± SD	—	—	14.36 ± 1.65
Gender	Male	137	54.8%
	Female	113	45.2%
Duration of diabetes	≤ 3 years	98	39.2%
	> 3 years	152	60.8%
Type of insulin therapy	Multiple daily injections	205	82%
	Insulin pump	45	18%
Smartphone ownership	Yes	189	75.6%
	No	61	24.4%
Internet access at home	Yes	130	52%
	No	120	48%
Family history of diabetes	Yes	54	21.6%
	No	196	74.8%
Mother's education	High school or less	63	25.2%
	University or higher	187	78.4%
Father's education	High school or less	89	35.6%
	University or higher	161	64.4%
Place of residence	Urban	162	64.8%
	Rural	88	35.2%
Perceived family socioeconomic status	Low	42	16.8%
	Middle	173	69.2%
	High	35	14.0%

**Fig. 1:**
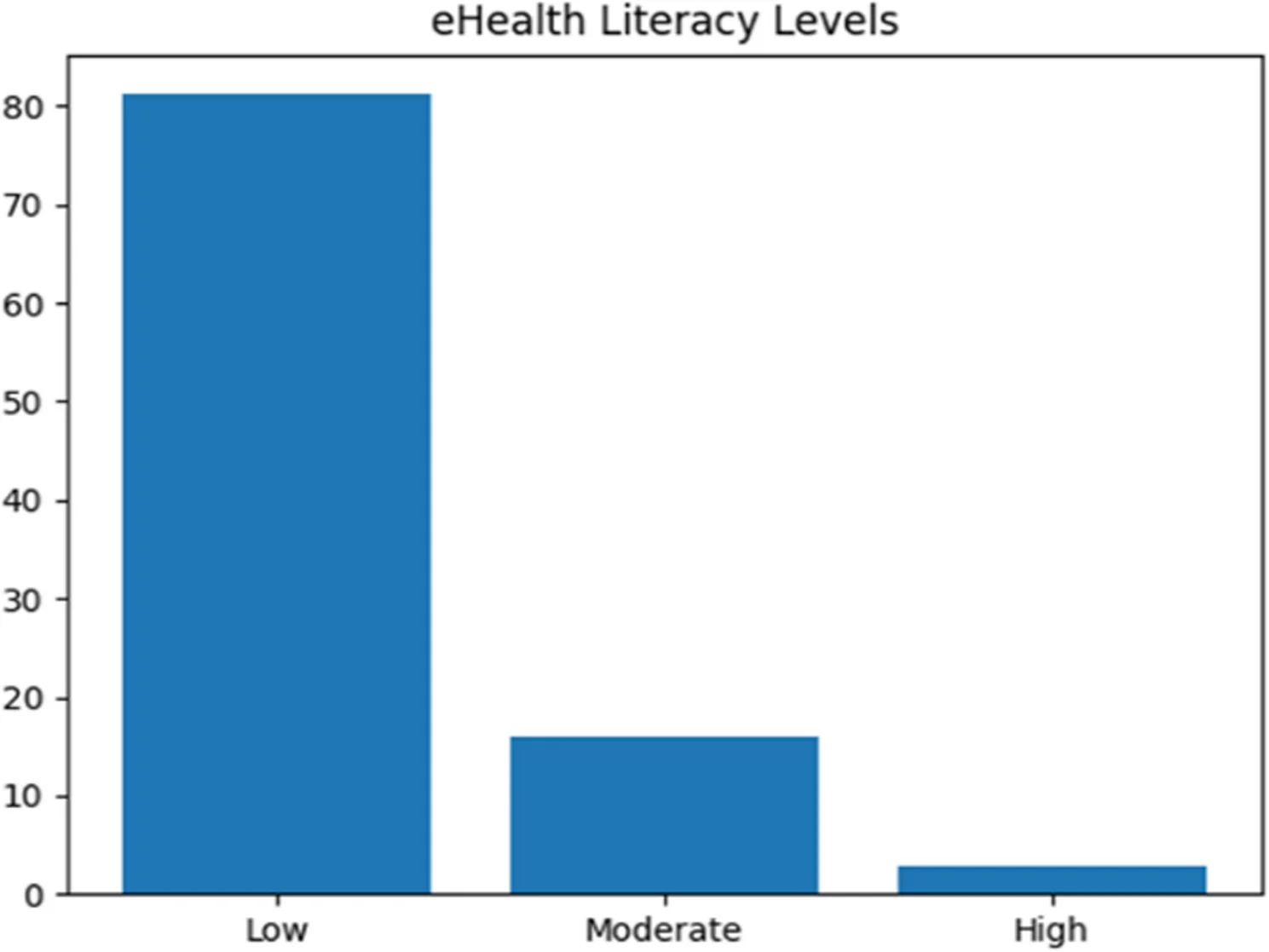
Bar chart of eHealth literacy levels in Adolescents with Type 1 Diabetes

**Fig. 2:**
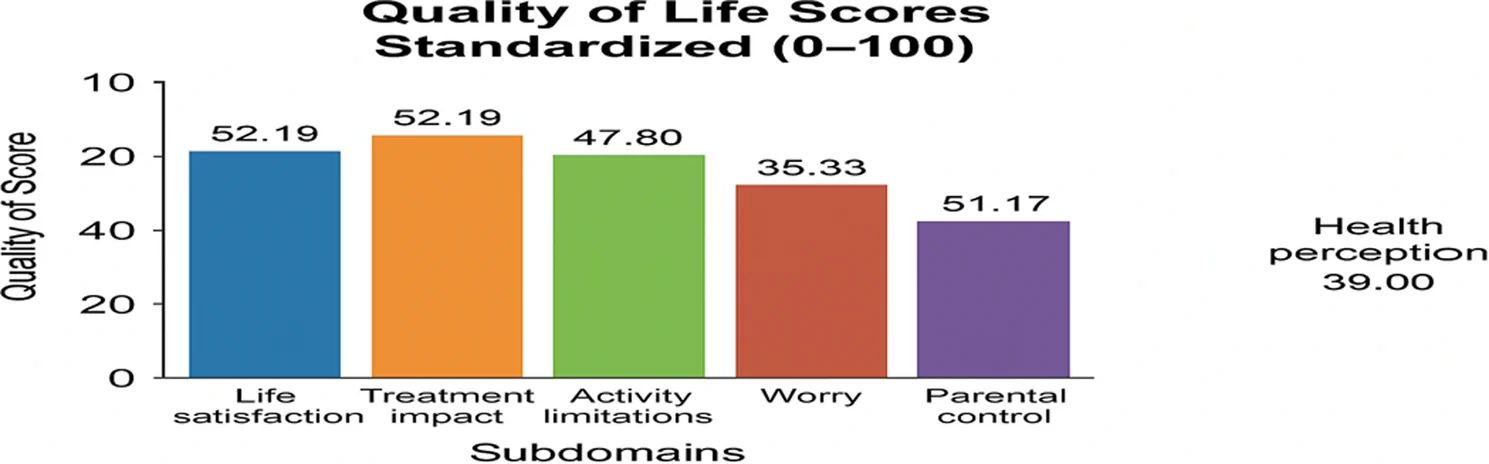
Standardized Quality of Life Scores Across Subdomains in Adolescents with Type 1

The mean total eHealth literacy score was 18.09 ± 6.95; most adolescents were classified as having low eHealth literacy (81.2%), followed by moderate (16.0%) and high literacy (2.8%) (Fig. 1), according to Rasouli et al. [11]. The mean total self-care score (SMOD-A) was 126.82 ± 41.91. Among the five self-care domains, the highest scores were observed in self-care activities, problem-solving, and collaboration with parents, whereas diabetes-related communication and diabetes goals showed the lowest scores. Health-related quality of life (DQOLY) had a total mean score of 83.27 ± 45.01, with the lowest subdomain in worries and the highest in treatment impact (Fig. 2).

As shown in Table 3, family socioeconomic status was significantly associated with eHealth literacy, self-care, and HRQoL (*p* < 0.05), while gender and mother’s education showed no significant differences. These findings highlight that adolescents from higher socioeconomic status backgrounds tend to have better self-care behaviors and quality of life.

**Table 3:** Group comparisons of eHealth literacy, self-care behaviors, and HRQoL by selected demographic variables (*n* = 250)

Variable	Category	*n*	eHealth Literacy (mean ± SD)	p-valueeHealth Literacy	Self-Care (mean ± SD)	*p*-valueSelf-Care	HRQoL (mean ± SD)	*p*-valueHRQoL
Gender	Male	137	32.5 ± 6.1	0.12*	78.3 ± 12.5	0.10*	71.4 ± 14.2	0.08*
	Female	113	31.8 ± 5.9		76.7 ± 13.1		70.1 ± 13.8	
Family SES	Low	42	31.0 ± 5.8	0.03**	74.5 ± 13.0	0.02**	69.8 ± 13.7	0.03**
	Middle	173	32.2 ± 6.1		77.3 ± 12.5		70.9 ± 14.0	
	High	35	33.1 ± 6.0		79.5 ± 12.1		72.5 ± 14.2	
Mother’s Education	High school or less	63	31.5 ± 5.9	0.08*	76.8 ± 12.7	0.09*	70.0 ± 14.0	0.07*
	University or higher	187	32.4 ± 6.1		77.9 ± 12.4		71.2 ± 14.0	

*Mann–Whitney U test, **Kruskal–Wallis test, *p* < 0.05 was considered statistically significant

**Fig. 3:**
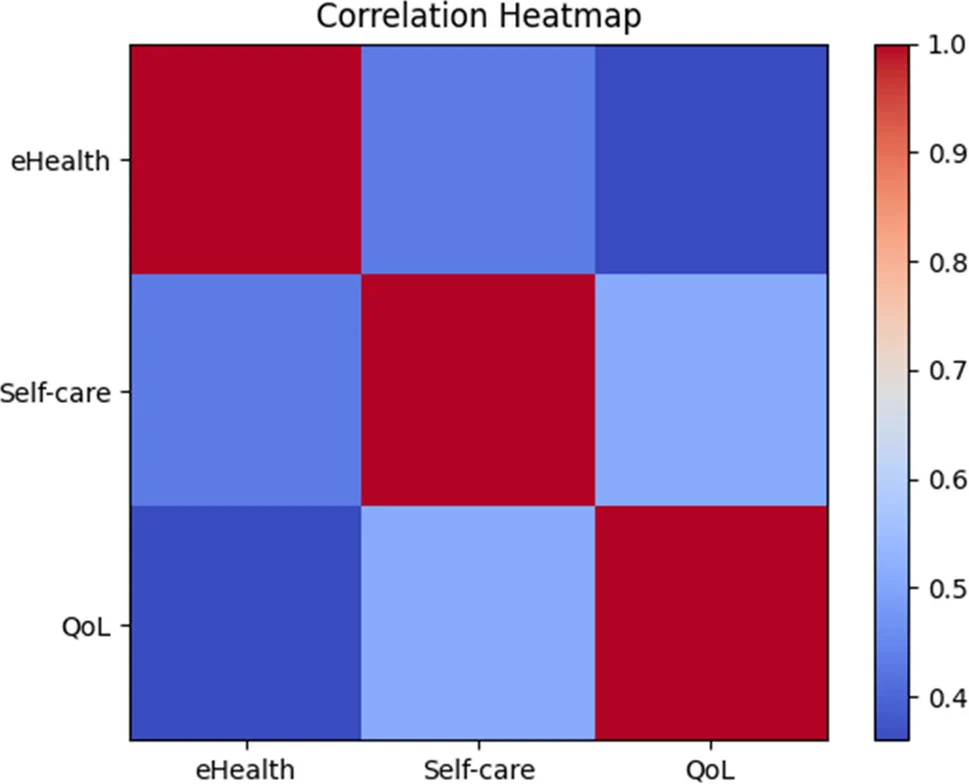
Correlation Heatmap of eHealth Literacy, Self Care, and HRQoL. Warmer colors indicate stronger positive correlations. The heatmap visually summarizes the associations reported in Table 4

Spearman’s correlation analysis revealed significant positive relationships among key variables. eHealth literacy correlated moderately with self-care behaviors (*r* = 0.43, *p* < 0.001) and weakly to moderately with quality of life (*r* = 0.36, *p* = 0.002). Self-care behaviors showed a stronger positive correlation with quality of life (*r* = 0.51, *p* < 0.001) (Table 4). Figure 3 presents a heatmap of the correlations among eHealth literacy, self-care, and quality of life, providing a visual summary of the associations reported in Table 4.

**Table 4:** Correlations Between eHealth Literacy, Self-Care Behaviors, and HRQoL

Variables	r	*p*-value	Interpretation
eHealth literacy ↔ Self-care	0.43	< 0.001	Moderate positive
eHealth literacy ↔ QoL	0.36	0.002	Weak-to-moderate
Self-care ↔ QoL	0.51	< 0.001	Moderate to strong

Spearman’s rank correlation coefficient, *p* < 0.05 was considered statistically significant

**Fig. 4:**
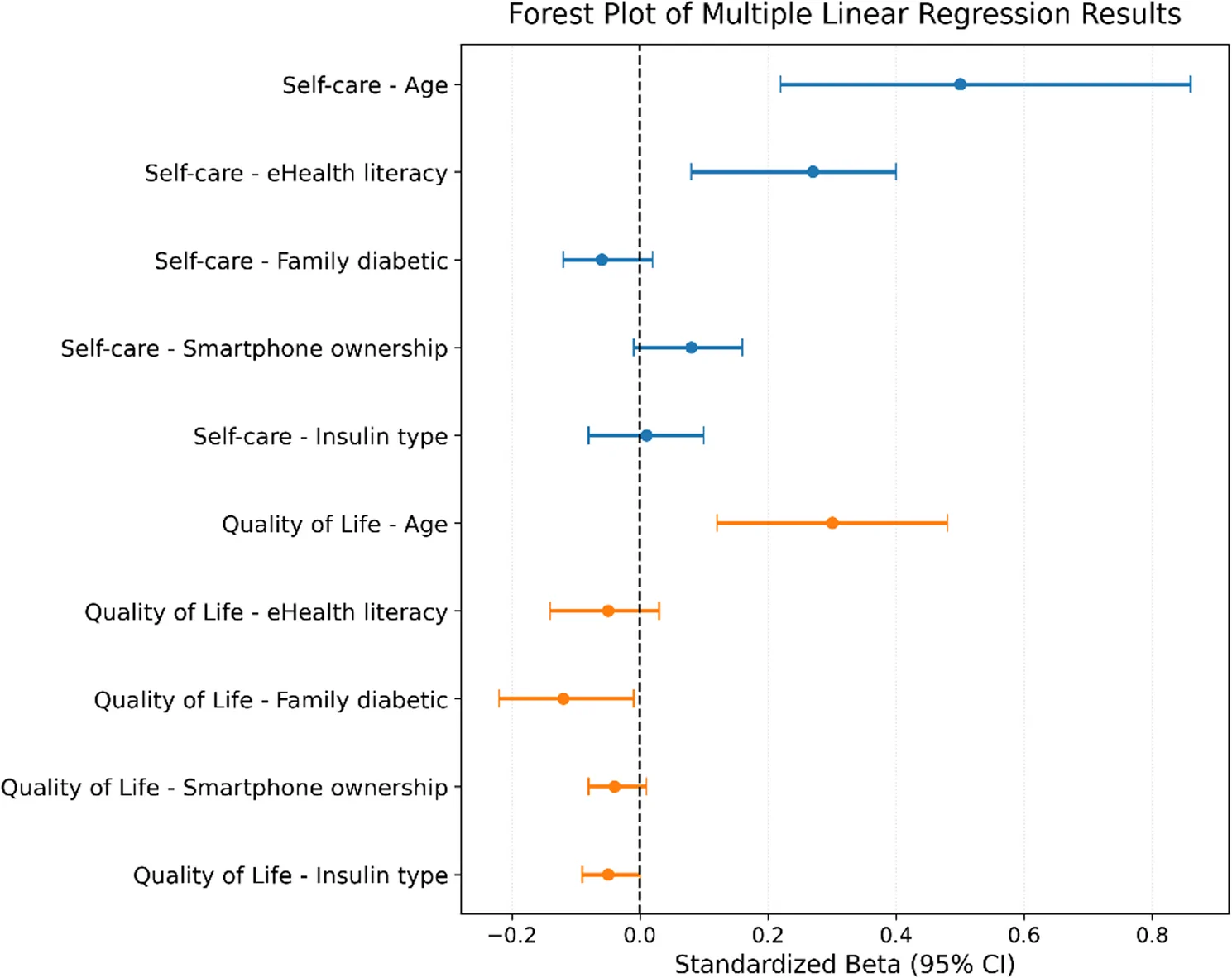
Forest plot of standardized beta coefficients from the multiple linear regression models for self-care behaviors and health-related quality of life. Standardized beta coefficients with 95% confidence intervals are presented for each predictor. The vertical line at zero represents no effect; confidence intervals crossing zero indicate non-significant associations

As shown in Table 5, Multiple linear regression analysis indicated that age (β = 0.546, *p* < 0.001) and eHealth literacy (β = 0.245, *p* < 0.001) were significant predictors of self-care behaviors. For quality of life, age (β = 0.312, *p* < 0.001) and family history of diabetes (β = − 0.138, *p* = 0.021) were significant predictors, whereas eHealth literacy was not a significant independent predictor. The positive association between eHealth literacy and self-care behaviors is illustrated in Fig. 4, where higher eHealth literacy scores are associated with improved self-care performance.

**Table 5:** Multiple Regression Analysis for Predictors of Self-Care and Quality of Life (*n* = 250)

Predictor	β (Self-care)	p-value	β (QoL)	p-value
Age (SEN)	0.546**	<0.001	0.312**	<0.001
eHealth literacy	0.245*	<0.001	–0.051	0.317
Family member diabetic	–0.063	0.172	–0.138*	0.021
Smartphone ownership¹	0.074	0.103	–0.032	0.092
Insulin injection type²	0.010	0.831	–0.041	0.084

All regression models were statistically significant (*p* < 0.001)

^1^ Smartphone ownership: 1 = Yes, 0 = No

^2^ Insulin injection type: 1 = Insulin pump, 0 = Pen

*p < 0.05, **p < 0.001

The results of the multiple linear regression models are summarized visually in Fig. 4. For the self-care model, older age and higher eHealth literacy were significantly associated with better self-care performance, while other predictors showed non-significant effects. For the quality-of-life model, age demonstrated a significant positive association, whereas family history of diabetes was significantly associated with lower quality of life. In contrast, eHealth literacy did not show a significant independent contribution to quality of life. The forest plot illustrates the magnitude and direction of effects across all predictors, where confidence intervals crossing zero indicate predictors without statistically significant contributions.

Mediation analysis demonstrated that self-care significantly mediated the relationship between eHealth literacy and HRQoL. As shown in Table 6, the estimated indirect effect of eHealth literacy on HRQoL via self-care was 0.219, indicating a significant mediation, whereas the direct effect of eHealth literacy on HRQoL was 0.141 and non-significant. These results suggest that higher eHealth literacy is associated with better self-care behaviors, which in turn contribute to improved quality of life.

**Table 6:** Mediation Analysis of Self-Care Between eHealth Literacy and Quality of Life (*n* = 250)

Path	Standardized Effect	Interpretation
eHealth literacy → Self-Care	0.43	Significant positive association
Self-Care → Quality of Life	0.51	Significant positive association
Indirect effect (eHealth → Self-Care → QoL)	0.219	Significant mediation
Direct effect (eHealth → QoL)	0.141	Non-significant

**Fig. 5:**
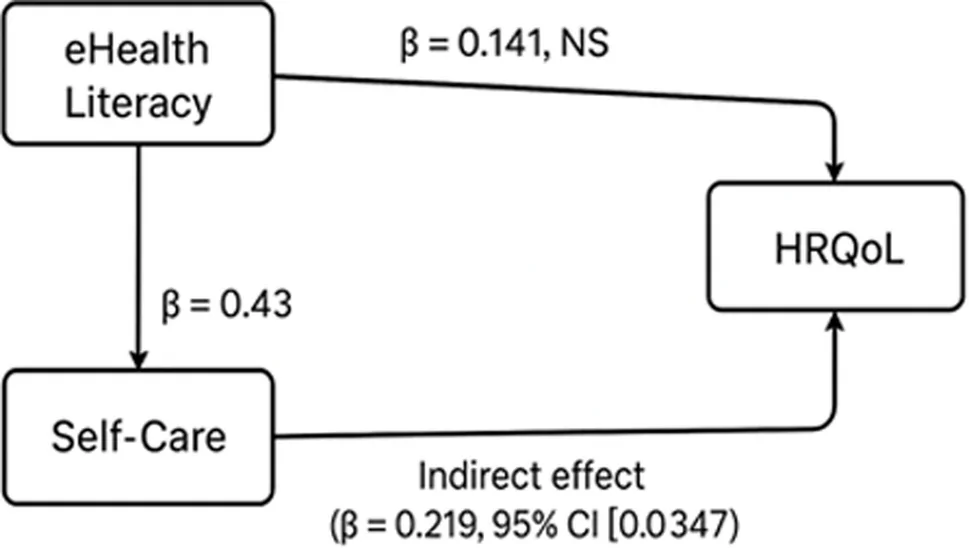
Path diagram of the mediation model showing direct and indirect effects ofeHealth literacy on HRQoL via self-care. Note: Standardized coefficients are displayed on the arrows

For a clearer understanding of the mediation model, Fig. 5 illustrates the path diagram, depicting both the direct and indirect effects along with their standardized coefficients. The visual representation allows readers to quickly grasp the structure of the model and the relative magnitude of the effects.

## Discussion

The present study investigated the determinants of self-care behaviors and health-related quality of life (HRQoL) among adolescents navigating type 1 diabetes. Multiple linear regression analysis elucidated that both age and eHealth literacy serve as significant predictors of self-care behaviors; specifically, older adolescents and those possessing superior competencies in navigating digital health information reported enhanced self-care engagement [8]. Conversely, while HRQoL demonstrated significant associations with age and familial history of diabetes, eHealth literacy failed to emerge as an independent predictor of HRQoL after adjusting for confounding variables [15]. This finding is consistent with previous studies suggesting that digital health literacy often exerts its influence on quality of life indirectly through behavioral mechanisms such as self-management and treatment adherence rather than through a direct association with HRQoL.

This observation is congruent with existing literature suggesting that eHealth literacy predominantly influences proximal outcomes, such as self-management, whereas its impact on distal outcomes like HRQoL is typically indirect and mediated through other behavioral mechanisms [16, 17]. Mediation analysis further revealed that self-care behaviors significantly facilitate the relationship between eHealth literacy and HRQoL. Adolescents with robust eHealth literacy were more likely to engage in effective self-care, which subsequently translated into superior quality of life. The direct effect of eHealth literacy on HRQoL was negligible and statistically non-significant, thereby underscoring the pivotal role of self-care as a primary intermediary mechanism. These findings extend the current empirical framework. by demonstrating that self-care serves as a critical bridge connecting digital health literacy to overall well-being [18].

Several studies from different contexts have reported findings similar to ours, suggesting that the influence of eHealth literacy on health outcomes is largely mediated through self-management behaviors. For instance, research in Palestine highlighted that general health literacy and self-efficacy are instrumental in optimizing self-care among adolescents with type 1 diabetes [18]. Similarly, studies from South Korea indicated that digital health literacy enhances HRQoL in diabetic populations by bolstering self-efficacy and self-care [19]. These findings closely align with our results, which also suggest that self-care behaviors act as a key pathway linking digital health literacy to adolescents’ quality of life. However, unlike some studies that reported a direct relationship between eHealth literacy and HRQoL, our analysis indicates that this association becomes non-significant after adjustment, highlighting the mediating role of self-care behaviors.Furthermore, German studies have noted that adolescents often encounter barriers when interacting with digital health interventions, suggesting that disparities in eHealth literacy may exacerbate differences in self-care engagement [8, 20]. Collectively, these insights emphasize that eHealth literacy likely influences quality of life through the sequential enhancement of self-care competencies.

Despite the predictive architecture of our mediation model, the cross-sectional design precludes definitive causal inferences. It is plausible that a bidirectional relationship exists: adolescents with advanced self-management skills may more proactively seek and critically evaluate digital health resources, thereby incrementally augmenting their eHealth literacy over time [21]. Consequently, while fostering eHealth literacy may catalyze better self‑care, this relationship should be interpreted with caution. It is also plausible that adolescents who are already more confident in their self‑management skills may be more motivated and adept at seeking credible online health information, gradually enhancing their eHealth literacy through active engagement. Longitudinal and interventional research will be critical to disentangle these potentially reciprocal pathways and confirm the temporal ordering between digital health competencies and behavioral outcomes.

A notable methodological consideration involves the use of the SMOD-A instrument. While this tool provides a comprehensive overview of self-management, the self-care construct it captures reflects a broad, global domain rather than discrete diabetes-specific behaviors. Consequently, it may lack the granularity needed to assess nuanced tasks such as precise insulin titration, frequency of blood glucose monitoring, or dietary adherence [22, 23]. Given this measurement structure, the mediating role observed in our model should be interpreted with the understanding that SMOD-A reflects general self-management competence rather than fine-grained diabetes behaviors.This limitation might obscure certain intricacies in the interplay between eHealth literacy and specific clinical behaviors. Future research should, therefore, triangulate self-reported data with objective clinical markers (e.g., continuous glucose monitoring data or HbA1c levels) and disease-specific scales to achieve a more nuanced assessment of this construct.

The observed inverse relationship between familial history of diabetes and HRQoL may reflect the deleterious impact of secondary trauma and the psychological burden associated with chronic illness [16, 17]. Adolescents’ proximity to the complications experienced by family members may exacerbate anxiety regarding their own disease progression, thereby diminishing their perceived quality of life.These findings suggest that eHealth literacy is a salient component in the supportive care of adolescents with type 1 diabetes. However, given the potential for bidirectionality, eHealth literacy interventions should not be implemented in isolation. Instead, they should be integrated into a holistic empowerment framework that combines digital literacy with practical self-care training. Synergizing eHealth education with clinical or school-based self-management programs may optimize both behavioral and psychosocial outcomes. Nevertheless, the efficacy of such multimodal approaches warrants further validation through longitudinal trials and formal mediation analyses [8, 18, 24].

### Study strengths and limitations

The strengths of this study include a robust sample size and the utilization of validated psychometric instruments. However, several caveats must be acknowledged:

1. The cross-sectional nature of the data limits the ability to establish causality.
2. Reliance on self-reported measures may introduce social desirability or recall bias.
3. Selection bias, stemming from recruitment at a single clinical center, may constrain the generalizability of the results.
4. The study lacked the statistical power for nuanced subgroup or interaction analyses; larger, multi-center cohorts are needed to explore demographic variations.
5. The SMOD-A tool, while comprehensive, provides a global overview of self-management but may lack diabetes-specific granularity (e.g., insulin titration, frequency of glucose monitoring). This limitation could have constrained the precision of the self-care construct used as a mediating variable [22, 23].

## Conclusion

In conclusion, this study suggests that eHealth literacy may indirectly foster improved quality of life in adolescents with type 1 diabetes by enhancing self-care behaviors. Due to the cross-sectional design, these results should be interpreted with academic caution. Integrating eHealth literacy into standard diabetes care—through clinical, educational, or digital platforms—represents a promising strategy, particularly when paired with hands-on self-management training. Future research employing longitudinal designs and objective clinical indicators will be essential to clarify these causal pathways and solidify the evidence regarding the mediating role of self-care.

## Data Availability

All data generated or analyzed during this study are included in this published article.
